# MyD88-adaptor protein acts as a preventive mechanism for memory deficits in a mouse model of Alzheimer's disease

**DOI:** 10.1186/1750-1326-6-5

**Published:** 2011-01-14

**Authors:** Jean-Philippe Michaud, Karine L Richard, Serge Rivest

**Affiliations:** 1 Laboratory of Endocrinology and Genomics, CHUL Research Center and Department of Molecular Medicine, Faculty of Medicine, Laval University, 2705 Laurier boul., Québec, G1V 4G2, Canada

## Abstract

**Background:**

Alzheimer's disease (AD) is an age-related neurodegenerative disorder associated with brain innate immune activation mainly mediated by microglia. These cells are known to be activated in the brain of AD patients and to produce inflammatory cytokines and neurotoxic molecules in response to Amyloid beta (Aβ). Activation of microglia can also promote Aβ clearance via Toll-like receptors (TLRs). Myeloid differentiation factor 88 (MyD88) is the adaptor molecule for most of these innate immune receptors, transducing the intracellular signal from TLRs to nucleus.

**Results:**

Here, we report that more than 50% reduction in MyD88 expression in a mouse model of AD accelerated spatial learning and memory deficits. Brain of APP_swe_/PS1-MyD88^+/- ^mice was characterized by a delay in accumulation of Aβ plaques and increased soluble levels of Aβ oligomers. Furthermore, inflammatory monocyte subset and brain IL-1β gene expression were significantly reduced in APP_swe_/PS1 mice with impaired MyD88 signaling.

**Conclusions:**

These data indicate that activation of MyD88 intracellular signaling pathway, likely by TLRs, acts as a natural innate immune mechanism to restrict disease progression of APP_swe_/PS1 mice.

## Introduction

Alzheimer's disease (AD) is the most common neurodegenerative pathology related to aging that results in progressive neuronal death and memory loss. Neuropathologically, this disease is characterized by the presence of both neurofibrillary tangles and plaques composed of aggregates of amyloid-β (Aβ) protein, a proteolytic fragment derived from the amyloid precursor protein (APP). As described by the amyloid cascade hypothesis, AD pathogenesis is associated with alterations in Aβ homeostasis resulting in an accumulation of Aβ peptides within the brain parenchyma that may exert neurotoxic effects and neuronal death [1]. Historically, amyloid plaques were correlated with cognitive deficits. Accumulating evidences suggest that soluble, oligomeric and even intracellular, rather than insoluble deposits of Aβ, correlate more strongly with dementia severity [2].

Inflammation is an important pathological component in the brains of AD patients. As proposed by the inflammation hypothesis of AD, the neurodegenerative process could be exacerbated by a chronic inflammatory response to Aβ peptides. Fibrillar Aβ deposits in AD brains are accompanied by innate immune responses such as activated microglia and increased levels of cytokines [1]. Microglia are the brain's tissue macrophages and the primary immune effectors within the CNS that respond to many pathological events. They are highly dynamic and respond rapidly to perturbations within the brain [3,4]. Accumulating evidence supports the hypothesis that microglia responses play a pivotal role in the pathogenesis of AD; however, their exact roles have not been yet resolved. Microglial activation is associated with production of neurotoxic mediators and inflammatory cytokines that can lead to neuronal death and may contribute to chronic neurodegenerative conditions [5]. On the other hand, microglia can phagocyte Aβ, produce Aβ degrading enzymes and then be neuroprotective by clearing Aβ from the brain [6]. The molecular mechanisms by which the innate immune system modulates the progression of AD are not well understood. A better understanding of the processes that regulate microglial activation may enhance the possibility of finding therapeutic approach against AD.

Toll-like receptors are transmembrane pattern recognition receptors that act as sensor for specific elements termed as pathogen-associated molecular pattern (PAMPs). These receptors are involved in innate immunity and inflammatory response by triggering activation of immune cells. To date, 10 different functional TLRs have been identified in humans and 12 in mice [7]. They display distinct ligand specificities and they are known to bind many endogenous proteins [8]. Recent work has implicated these receptors in microglial recognition and response to Aβ. Plaques associated-microglia exhibit elevated TLR2, TLR4, TLR5, TLR7 and TLR9 mRNA levels [9]. Stimulation of microglia with innate immune ligands for TLR2, TLR4 and TLR9 was found to accelerate Aβ clearance *in vitro *and in mouse model of AD [10,11]. Mutations in TLRs, such as TLR4, are risk factors for AD [12]. Aβ can also directly bind TLR2 and TLR4 [13].

Taken together these studies argue for the involvement of innate immune system and TLRs in the course of AD, although the exact mechanisms mediating these effects have yet to be uncovered. MyD88 bridges most TLRs to the downstream signaling elements mitogen-activated protein kinases (MAPKs) and nuclear factor-kappaB (NF-κB), which trigger transcriptional activation of inflammatory genes [8]. MyD88 is also essential for triggering expression of innate immune genes in microglia following exposure to both exogenous and endogenous toxic stimuli [14,15]. The aim of the present study was therefore to determine the role of MyD88 in the pathogenesis of AD. We thus generated a mouse model of AD in a context of MyD88 deficiency. Our data from these mice suggest a critical neuroprotective role of the MyD88 pathway in APP_swe_/PS1 mice.

## Materials and methods

### Transgenic mouse lines

APP_swe_/PS1 transgenic mice harbouring the human presenilin I (A246E variant) and the chimeric mouse/human Aβ precursor protein (APP695_swe_) under the control of independent mouse prion protein (PrP) promoter elements ([B6C3-Tg(APP695)3Dbo Tg(PSEN1)5Dbo/J]; Jackson Laboratories) were bred with MyD88^-/- ^mouse strain for at least three generations to generate APP_swe_/PS1 mice heterozygous for the MyD88 gene. MyD88^-/- ^mice (in C57BL/6 background) were provided by S. Akira (Osaka University, Osaka, Japan). All newborn pups were genotyped and included in the different experimental groups. All mouse strains were maintained in a C57BL/6J background and acclimated to standard laboratory conditions. All protocols were conducted according to the Canadian Council on Animal Care guidelines, as administered by the Laval University Animal Welfare Committee.

### Fluorescent-activated cell sorting (FACS) analysis

To analyze the population of monocytes, anti-coagulated whole blood was taken from the facial vein, quickly suspended and cells were washed with DPBS with 4% of fetal bovine serum (FBS). Cells were then re-suspended into DPBS with 2% FBS and incubated on ice with purified rat anti-mouse CD16/CD32 (Mouse BD Fc Bloc, BD Bioscience). Still on ice, the mix was incubated with PE-Cy7 conjugated CD11b antibody (eBioscience), APC conjugated CD115 antibody (eBioscience), PerCP cy5.5 conjugated Gr1 antibody (Cerdarlane) and CD45 PE-Cy5 (BD Bioscience) for 45 minutes. Cells were washed again in DPBS with 2% FBS. Red blood cells were lysed with hemolysin according to manufacturer's protocol (Beckman Coulter, Mervue, Galway, Ireland) and cells were washed with DPBS and re-suspended in equal volumes of DPBS. The cells were analyzed using a two-lasers and six color FACS Canto II flow cytometer and data acquisition was done with BD Facs Diva software (Version 6.1.2, BD Biosciences, San Jose, CA). Cells were sorted according to the different fluorescent antibodies. Results were analyzed using Flow Jo software (Tristar).

### Tissue collection

To collect brain tissues for immunofluorescence, mice were deeply anesthetized via an i.p. injection of a mixture of ketamine hydrochloride and xylazine and then were perfused intracardially with ice-cold 0.9% saline, followed by 4% paraformadehyde in a 0.1 M borax buffer (pH 9.5 at 4°C). Brains were rapidly removed from skulls, post-fixed in PFA 1-3d at 4°C and cryoprotected in 10% sucrose diluted in PFA overnight. The frozen brains were then sectioned into 25-μm thick coronal sections using a microtome (Cambridge Instruments Company) and slices were collected in a cold cryoprotectant solution (0.05M sodium phosphate buffer, pH 7.3, 30% ethylene glycol, 20% glycerol, stored at -20°C). For protein analysis, brains were decapitated, rapidly removed from the skull and frozen in liquid nitrogen. They were subsequently stored at -80°C until protein extraction and detection.

### Stereological analysis

To stain Aβ plaques, free-floating sections were treated 30 min with a permeabilization/blocking solution containing 0.4% Triton X-100, 4% goat serum and 1% bovine serum albumin (BSA; Sigma-Aldrich) in KPBS. Using the same solution, sections were immunostained 60 min with monoclonal anti-Aβ (6E10, 1:3,000; Chemicon international), washed three times for 5 min in KPBS and then incubated with secondary antibody goat anti-mouse Cy3 conjugated (1:1,000; Jackson Immunoresearch Laboratories, inc) for 60 min. Stereological analysis was performed as described previously [16]. The contours of the cortex and hippocampus areas were traced as virtual overlay on the steamed images and areas was calculated. For 3 and 6-month-old APP mice, the area occupied by all Aβ-labeled plaques was determined. For the analysis of 9-month-old APP mouse plaques, the Stereo Investigator software (Microbrightfield) sequentially chose counting frame in cortex and hippocampus. Areas of analyzed hippocampal or cortex region were calculated as well as areas occupied by plaques in those counting frames.

### Protein extraction

Proteins from hemi-forebrains were extracted using a modified method of the procedure published by Lesne *et al*. [17]. All manipulations were done on ice to minimize protein degradation. One hemi-forebrain was placed in a 1 ml syringe with a 20 G needle. 500 μl of buffer A (50 mM Tris-HCl pH 7.6, 0.01% NP-40, 150 mM NaCl, 2 mM EDTA, 0.1% SDS, 1 mM phenylmethylsulfonyl fluoride (PMSF), protease inhibitor cocktail) were added and 10 up and down were made to homogenize the tissue, followed by a 5 minutes centrifugation at 3 000 RPM at 4°C. The supernatant (extracellular proteins enriched fraction) was then collected and frozen at -80°C. The insoluble pellet was suspended in 500 μl TNT-buffer (Buffer B; 50 mM Tris-HCl pH 7.6, 150 mM NaCl, 0.1% Triton X-100, 1 mM PMSF, protease inhibitor cocktail), followed by a 90 minutes centrifugation at 13 000 RPM at 4°C. The supernatant (cytoplasmic proteins enriched fraction) was then collected and frozen at -80°C. The pellet was suspended in 500 μl buffer C (50 mM Tris-HCl pH 7.4, 150 mM NaCl, 0.5% Triton X-100, 1 mM EGTA, 3% SDS, 1% deoxycholate, 1 mM PMSF, protease inhibitor cocktail) and incubated at 4°C, 50 RPM, for 1 hour. The samples were centrifuged for 90 minutes at 13 000 RPM and 4°C and the supernatant (membrane proteins enriched fraction) was collected and frozen at -80°C. Protein concentration of each fraction was determined using the Quantipro BCA assay kit (Sigma) according to the manufacturer protocol.

### Detection of total Aβ levels by Western blot

For total Aβ detection by Western blot, 10-30 μg of extracellular protein fractions were separated on a precast 10-20% SDS polyacrylamide Tris-Tricine gel (Bio-Rad). Separated proteins were then transferred onto polyvinylidene fluoride (PVDF) membranes (PerkinElmer Life and Analytical Sciences) and detected by Western blotting. Blots were probed with a mouse anti-amyloid beta protein monoclonal antibody clone 6E10 (1:1000, Covariance) in Tris-buffered saline with 0.05% Tween 20 (TBS-T) and 5% skim milk. Blots were visualized using anti-mouse secondary antibody tagged with horseradish peroxidase (1:1000; Jackson Immuno-Research) and ECL Plus Detection Reagents (GE Healthcare). Membranes were stripped in 25 mM glycine-HCl, pH 2.0, containing 1% SDS to allow β-actin revelation using first a mouse β-actin antibody (MAB1501, 1:10 000; Millipore Bioscience Research Reagents) and then a goat anti-mouse peroxidase conjugated secondary antibody (1:10 000; Jackson ImmunoResearch). Semi-quantitative analysis of signal was performed under a Northern Light Desktop Illuminator (Imaging Research) using a Sony Camera Video System and the NIH Image J software version 1.32J. Optical values were normalized according to the β-actin loading control.

### RNA extraction and quantitative real-time PCR (Q-PCR)

Tissue samples were homogenized in Qiazol buffer (Qiagen) and extracted using the RNeasy mini kit with on-column DNase (Qiagen) treatment following the manufacturer's recommendations. RNA concentration was measured using a NanoDrop spectrophotometer (Thermo Scientific) and quality was assessed with an Agilent 2100 Bioanalyzer (Agilent Technologies Inc). First-strand cDNA synthesis was accomplished using 5 μg of isolated RNA in a reaction containing 200 U of Superscript III Rnase H-RT (Invitrogen Life Technologies), 300 ng of oligo-dT_18_, 50 ng of random hexamers, 50 mM Tris-HCl pH 8.3, 75 mM KCl, 3 mM MgCl_2_, 500 μM deoxynucleotides triphosphate, 5 mM dithiothreitol, and 40 U of Protector RNase inhibitor (Roche Diagnostics) in a final volume of 50 μl. The reaction was performed at 25°C for 10 minutes following at 50°C for 1 hour and then treated with 1 μg of RNase A for 30 min at 37°C. The resulting products were purified with Qiaquick PCR purification kits (QIAGEN). cDNA corresponding to 20 ng of total RNA was used to perform fluorescent-based Realtime PCR quantification using the LightCycler 480 (Roche). The following sets of sense/antisense primers were used: for MyD88: 5'-TCCGGCAACTAGAACAGACAGACT-3'/5'-GCGGCGACACCTTTTCTCAAT-3', IL-1β: 5'- TCAAATCTCGCAGCAGCACATC-3'/5'-CCAGCAGGTTATCATCATCATCCC-3', 18S: 5'-TGGATACCGCAGCTAGGAATAATG-3'/5'-TCACCTCTAGCGGCGCAATAC-3', Hprt1: 5'-CAGGACTGAAAGACTTGCTCGAGAT-3'/5'-CAGCAGGTCAGCAAAGAACTTATAGC-3'. Reagent LightCycler 480 SYBRGreen I Master was obtained from the same company and was used as described by the manufacturer. The conditions for PCR reactions for 45 cycles were: denaturation at 95°C for 10 sec, annealing at 60°C for 10 sec and elongation at 72°C for 12 sec. The reaction was then heated for 5 sec at 2°C lower than the melting temperature of the DNA fragment. Reading of the fluorescence signal was taken at the end of the heating to avoid non-specific signal. A melting curve was performed to assess non-specific signal. Oligoprimer pairs that allow the amplification of approximately 200 bp were designed by GeneTools software (Biotools Inc) and their specificity was verified by blast in the GenBank database. Data calculation and normalization was performed using second derivative method as described previously [18] using the reference genes hypoxanthine guanine phosphoribosyl transferase 1 (Hprt1) and 18S ribosomal RNA (18S). PCR amplification efficiency was verified.

### Behavioral analysis

Mice were housed four or five per cage in a colony maintained in a room under natural lighting conditions with a 14/10 hr light-dark cycle (on at 6:00) and tested during the "light on" phase of the day. Food and water were provided *ad libitum*. Behavioral experimenter was blinded to the genotype of animals. The behavioral and general health of mice was monitored using a modified version of the primary observation screen described by the SHIRPA protocol [19]. The spatial learning abilities of mice were assessed in the T-water maze task (TWM). The T-maze apparatus (length of stem 64 cm, length of arms 30 cm, width 12 cm, height of walls 16 cm) was made of clear fiberglass and filled with water (23 ± 1°C) at a height of 12 cm. An escape platform (11×11 cm) was placed at the end of the target arm and was submerged 0.5 cm below the surface. The acquisition phase allows to evaluate animals for left-right spatial learning. During the first two trials, platforms were placed on each arms of the TWM. In order to assess arm preferences, the least chosen arm was reinforced by water escape. The mice were placed in the stem of the T maze and choose to swim either left or right until they found the submerged platform and escape to it, to a maximum of 60 s. After reaching the platform, the mice were allowed to stay on it for 20 s and then were immediately placed back in the maze. If the animals did not find the platform within this limit, they were gently guided onto it. Repeated trials were presented on the same day up to a maximum of 48 trials. A rest period of at least 10-15 min intervened between each block of 10 trials. A mouse was considered to have learned the task when it made no errors in a block of five consecutive trials. The reversal learning phase was then conducted 48 hours later. During this phase the same protocol was repeated, except that the mice were trained to find the escape platform on the opposite side to that on which they had learned on acquisition phase. Two measures were taken: number of trials to reach the criterion (5 of 5 correct choices made on consecutive trials) and escape latency.

### Statistical analysis

All statistical analysis was performed using the SPSS software (SPSS 15.0). Homogeneity of variance in our different samples was performed with the Levene's test and *p *< 0.05 was set as the level of a significant difference.

## Results

### Reduced expression of MyD88 adaptor protein in APP_swe_/PS1 mice increased memory deficits

**Figure 1:**
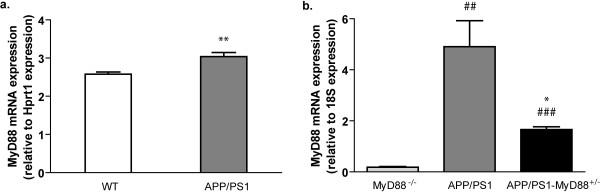
**Relative expression of MyD88 expression in the brain of different mouse strains**. **(a) **MyD88 mRNA expression is upregulated in 6 month-old APP_swe_/PS1 mice compared to WT littermates. ** *P *< 0.05 (vs WT) **(b) **APP_swe_/PS1-MyD88^+/- ^mice expressed significantly lower level of MyD88 mRNAs compared to APP_swe_/PS1 mice at 3 months of age. ## *P *< 0.01, ### *P *< 0.001 (vs MyD88^-/-^); * *P *< 0.05 (vs APP_swe_/PS1). Data are expressed as mRNA arbitrary units normalized with housekeeping genes Hprt1 and 18S. Error bars: SEM; n = 3-4. (Statistical analysis was performed using Student's *t*-test in **(a) **and a one-way ANOVA followed by a Bonferroni *post-hoc *test in **(b)**).

As the brain tissue macrophage, microglia expresses a wide range of TLRs to detect exogenous and endogenous ligands. It is now well known that microglia use these receptors to recognize and phagocyte Aβ peptides. We and others previously demonstrated that the TLR2 is necessary for the microglia to mount an inflammatory reaction against Aβ [13,20,21]. When stimulated, TLRs activate intracellular signaling cascade via the adaptor protein MyD88. We show here that MyD88 mRNA expression is upregulated in response to Aβ (Figure 1a). Indeed, quantitative real-time PCR (Q-PCR) analysis of 6 month-old APP_swe_/PS1 mice revealed a significant increase in MyD88 mRNA compared to WT littermates. To study the role of this major signaling protein, we attempted to breed the APP_swe_/PS1 mouse with the MyD88 knockout mice, which share the same background. Unfortunately, APP_swe_/PS1 mice lacking the MyD88 adaptor protein were not viable. APP_swe_/PS1 mice heterozygous for the MyD88 gene were therefore used for further analyses. By 3 months of age, MyD88 mRNA levels in the brain of APP_swe_/PS1-MyD88^+/- ^mice were reduced of 66% compared to those of APP_swe_/PS1 mice as determined by Q-PCR (Figure 1b). Arbitrary values obtained for APP_swe_/PS1 and APP_swe_/PS1-MyD88^+/- ^groups were 4.90 ± 1.02 and 1.66 ± 0.11. As expected, MyD88^-/- ^mice did not express any detectable MyD88 mRNA (arbitrary value: 0.18 ± 0.03).

**Figure 2:**
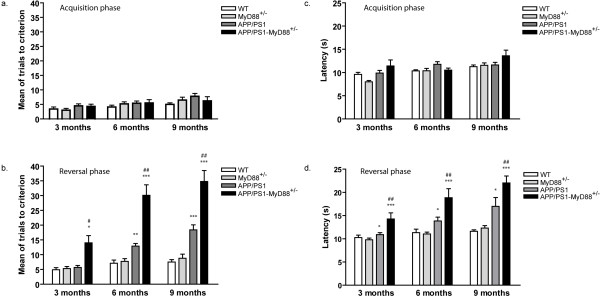
**Reduced expression of the adaptor protein MyD88 in APP_swe_/PS1 mice accelerates spatial memory deficits in the T-water maze paradigm**. Mice were trained to reach the hidden platform on one arm of the T-water maze apparatus. (**a**) Number of trials and time spent **(c) **to learn the task were measured. All different strains of each ages performed similarly. Spatial learning and memory of trained mice was challenged 48 hours later (Reversal phase). Compared with APP_swe_/PS1 mice and their controls, APP_swe_/PS1-MyD88^+/- ^transgenic mice showed a greater decline of spatial cognitive capacities measured by the number of errors (**b**) and time spent (**d**) to reach the platform. Error bars represent SEM; n = 8-11; * *P *< 0.05 (vs WT and MyD88^-/-^), ** *P *< 0.01 (vs WT and MyD88^-/-^), *** *P *< 0.001 (vs WT and MyD88^-/-^), # *P *< 0.05 (vs APP_swe_/PS1), ## *P *< 0.01 (vs APP_swe_/PS1), ### *P *< 0.001 (vs APP_swe_/PS1). (Statistical analysis was performed using one-way ANOVA followed by a Bonferroni or Tamhane's *post-hoc *test).

We next evaluated APP_swe_/PS1-MyD88^+/- ^mice in the T-water maze for behavioral evaluation. Hippocampus-based spatial learning and memory was assessed by the ability of mice to acquire (training session) and retrieve (test session) spatial information as indicative of learning and memory functions. Escape latency and mean of trials to reach the hidden platform placed on one arm of the maze were recorded. Mice that have found and climbed onto the hidden platform on five consecutive trials were considered to have reached the criterion. During the initial training session, all groups exhibited comparable performance, as demonstrate by the numbers of errors (Figure 2a) and time spent (Figure 2c) to learn the task. No significant differences in term of swimming performance or motivation were detected between each group of mice. We performed a probe trial 48 h after completing the initial training to evaluate the cognitive flexibility of our different mouse strains. The hidden platform was placed on the opposite arm of the T-maze apparatus and mice were placed back in the maze. Both wild-type and MyD88^+/- ^mice showed equally successful latency and number of trials to criterion during the reversal task. Because of their behavioral phenotype, APP_swe_/PS1 mice demonstrate signs of cognitive deficits at 6 and 9 months of age compared to controls. The decreased expression of MyD88 gene in APP_swe_/PS1 transgenic mice resulted in a significant decline in memory as indicated by longer latencies (Figure 2d) and increased number of errors committed (Figure 2b) to reach the platform during the probe trial, compared to results obtained for APP_swe_/PS1 mice. Those cognitive deficits began as early as 3 months (5.7 ± 0.6 trials and 11.0 ± 0.5 seconds for APP_swe_/PS1 vs 14.2 ± 2.4 trials and 14.0 ± 1.3 seconds for APP_swe_/PS1-MyD88^+/-^) and evolved rapidly at 6 (13.0 ± 1.0 trials and 13.5 ± 0.7 seconds for APP_swe_/PS1 mice vs 30.1 ± 3.5 trials and 18.9 ± 1.9 seconds for APP_swe_/PS1-MyD88^+/- ^mice) and 9 months (18.4 ± 1.7 trials and 17.0 ± 1.9 seconds for APP_swe_/PS1 vs 34.7 ± 3.7 trials and 22.0 ± 1.5 seconds for APP_swe_/PS1-MyD88^+/-^). These results demonstrate that the reduce expression of MyD88 in APP_swe_/PS1 mice result in a greater cognitive deficit as depicted by accelerated spatial memory impairment. These data suggest that MyD88 signaling plays an important role in microglia activation in order to fight against AD evolution.

### Aβ plaques accumulate slower in brains of APP_swe_/PS1-MyD88^+/- ^mice while more soluble Aβ is detected

**Figure 3:**
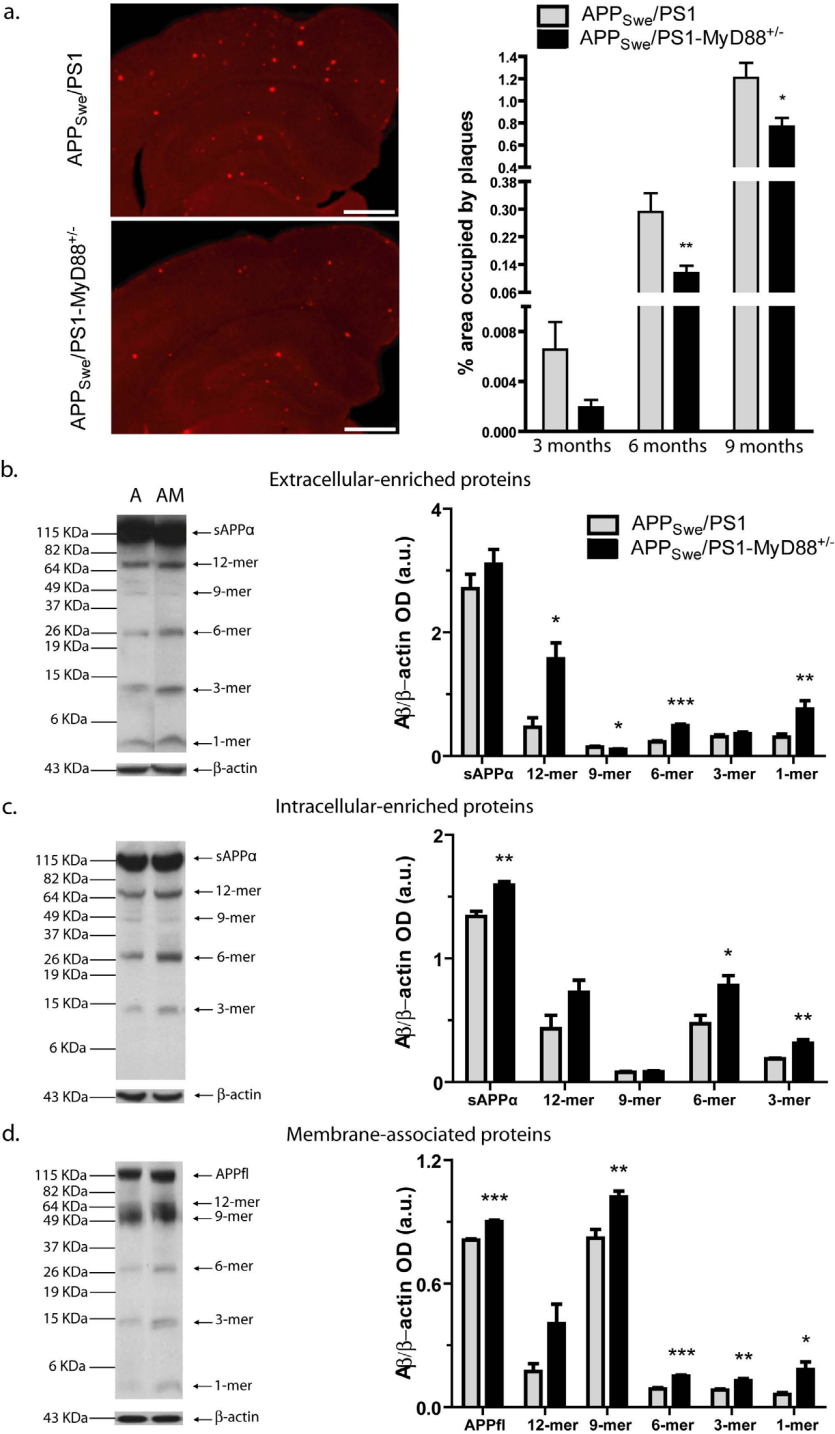
**Amyloid plaque load and soluble Aβ levels in the brain of APP_swe_/PS1 in a context of partial MyD88 deficiency**. Deposition of Aβ plaques is significantly more abundant in 6 and 9 month-old APP_swe_/PS1 compared to APP_swe_/PS1-Myd88^+/- ^mice **(a)**. Aβ immunoreactivity in cortex and hippocampus is shown in brain sections of 9 month-old APP_swe_/PS1 and APP_swe_/PS1-Myd88^+/- ^mice. Percentage of area covered by plaques was quantified for mice of 3, 6 and 9 month-old, respectively. n = 9-10. (Two-way ANOVA was performed revealing a significant interaction between factors age and genotype. The comparison of genotype for each age was performed by Student's *t*-test). To detect soluble Aβ, western blot analysis on 10-20% Tris-Tricine denaturing polyacrylamide gels of extracellular **(b)**, intracellular **(c) **and membrane-associated **(d) **enriched proteins of 6 month-old mice were assessed using monoclonal 6E10 antibody to reveal the different species. Most of Aβ oligomers were significantly higher in the brains of APP_swe_/PS1-MyD88^+/- ^(AM) mice than that of APP_swe_/PS1 (A) in all protein fractions. Bands depicted here were cut from the same membrane for each protein fraction. Values are expressed as optical densities (OD) in arbitrary units (a.u.) of Aβ normalized with β-actin. n = 4-7; Student's *t*-test; * *P *< 0.05, ** *P *< 0.01, *** *P *< 0.001. Error bars: SEM. Scale bar = 500 μm

As amyloid plaques are a neuropathological marker of AD, we compared amyloid plaque load in APP_swe_/PS1 and APP_swe_/PS1-MyD88^+/-^. Aβ immunoreactivity was measured with the monoclonal 6E10 antibody specific for the fragment 1-17 a.a. of the human Aβ peptide in brain coronal section. Area of Aβ deposits was evaluated in the cortex and hippocampus by stereological analysis in two similar sections per animal and percentage area occupied by plaques was calculated (Figure 3a). At three months of age, no statistically significant difference in area occupied by plaques was observed between APP_swe_/PS1 and in APP_swe_/PS1-MyD88^+/- ^mice (0.007 ± 0.002% and 0.002 ± 0.0006%, respectively). At six and nine months, Aβ deposits were significantly lower in APP_swe_/PS1-MyD88^+/- ^brains compared to their APP_swe_/PS1 littermates (0.292 ± 0.054 vs 0.116 ± 0.021, 1.208 ± 0.135 vs 0.769 ± 0.075, 6 and 9 month-old APP_swe_/PS1 and APP_swe_/PS1-MyD88^+/-^, respectively).

Since Aβ plaques did not correlate with cognitive deficits, we decided to measure soluble levels of Aβ species in brains of 6 month-old mice - there is accumulating evidence associating soluble Aβ oligomers with cognitive impairments [2,17,22,23]. The amounts of different Aβ species were analyzed in extracellular (Figure 3b), intracellular (Figure 3c) and membrane-associated (Figure 3d) enriched protein fractions by western blots, based on the method developed by Lesne *et al*. [17]. Proteins from wild-type animals were used as control to assess the specificity of the antibody and the signal was barely detectable in such a case (data not shown). In general, most Aβ oligomers were increased in the brain of APP_swe_/PS1-MyD88^+/- ^mice compared to that of APP_swe_/PS1. Indeed, there were significantly higher levels of Aβ oligomers in extracellular (1-, 6- and 12-mer), intracellular (3- and 6-mer) and membrane-associated (1-, 3-, 6-, 9-mer) enriched proteins fractions. Levels of sAPPα levels and APP full length (APPfl) were also significantly higher in brains of APP_swe_/PS1-MyD88^+/- ^mice in intracellular and membrane-associated enriched proteins, respectively. These data indicate that soluble oligomeric Aβ levels increased while the amyloid-β plaque load decreased in brains of AD mice that had down-regulated MyD88 gene expression.

### Inflammatory monocyte subset is decreased while resident monocytes are increased in the blood of APP_swe_/PS1-MyD88^+/- ^mice

**Figure 4:**
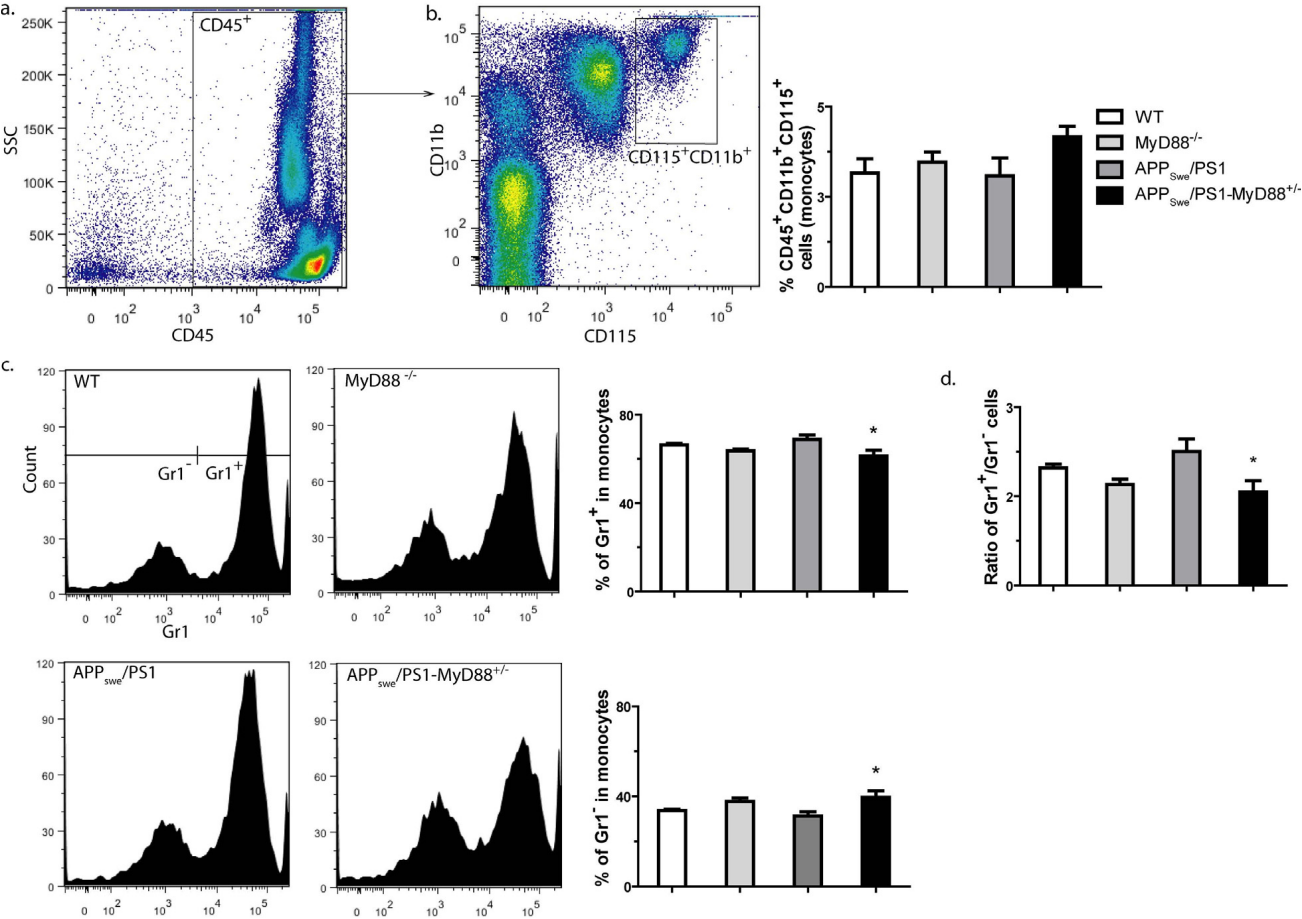
**Flow cytometry analysis of blood monocyte subsets in WT, MyD88^-/-^, APP_swe_/PS1 and APP_swe_/PS1-MyD88^+/- ^mice**. Blood taken from the facial vein of 3-4 month-old mice was analyzed by FACS with the following fluorescently-labeled antibodies: CD45, CD11b, CD115 and Gr1. Leukocyte population is represented as cells in the CD45^+ ^gate (**a**). To analyze exclusively monocytes in CD45^+ ^cells, CD11b^hi ^and CD115^hi ^(CD45^+^CD11b^+^CD115^+^) cells were gated and the total percent for each group was quantified (**b**). No significant change in number of monocytes was revealed. Representatives histograms of monocytes for each group and their mean relative Gr1^+ ^and Gr1^- ^values (**c**) showed significantly less Gr1^+ ^and more Gr1^- ^monocytes in APP_swe_/PS1-MyD88^+/- ^mice compare with APP_swe_/PS1 mice. This difference is even more noticeable while expressing the ratio of Gr1^+ ^on Gr1^- ^monocytes (**d**). Error bars: SEM; n = 3-5; *P < 0.05 (vs APP_swe_/PS1). (Statistical analysis was performed using one-way ANOVA followed by a Dunnett's or Tamhane's *post-hoc *test).

It has been established that there is two principal blood monocyte populations that possess different migratory properties: the "inflammatory subset" which is characterized by a CX_3_CR_1_^Lo^CCR2^+^Gr1^+ ^phenotype and the "resident subset" categorized by CX_3_CR_1_^Hi^CCR2^-^Gr1^- ^[24]. To evaluate if these two subsets of monocytes were different in animals with reduced MyD88 protein, we measured expression levels of Gr1 surface marker on monocytes of 3-4 month-old mice. To analyze leukocytes in blood cells, we included only cells that were positive for CD45 (Figure 4a). Then, CD45^+^CD11b^+^CD115^+ ^cells were selected and considered as the population of monocytes (Figure 4b). There was no significant difference between percentages of monocytes (Figure 4b) or CD45^+ ^cells (data not shown) among the 4 groups. Relative proportions of Gr1^+ ^and Gr1^- ^cells were then evaluated in the monocyte population (Figure 4c). There was significantly less Gr1^+ ^and more Gr1^- ^cells in APP_swe_/PS1-MyD88^+/- ^compared to APP_swe_/PS1 mice (61.27 ± 2.66% vs 68.80 ± 2.04% and 39.58 ± 2.81% vs 31.26 ± 2.04%, respectively). To evaluate the global difference between these two populations of monocytes, the ratios of Gr1^+ ^monocytes on Gr1^- ^monocytes were calculated (Figure 4d). These ratios revealed a significant decrease for APP_swe_/PS1-MyD88^+/- ^mice compared to their APP_swe_/PS1 control (1.57 ± 0.19% vs 2.25 ± 0.21%, respectively). These results suggest that MyD88 pathway has a significant impact on blood monocyte populations in this mouse model of AD, which exhibits increased amount of the resident subset and a decreased level of inflammatory monocytes in a context of partial MyD88 deficiency.

### IL-1β gene expression is decreased in the brain of APP_swe_/PS1-MyD88^+/- ^mice

**Figure 5:**
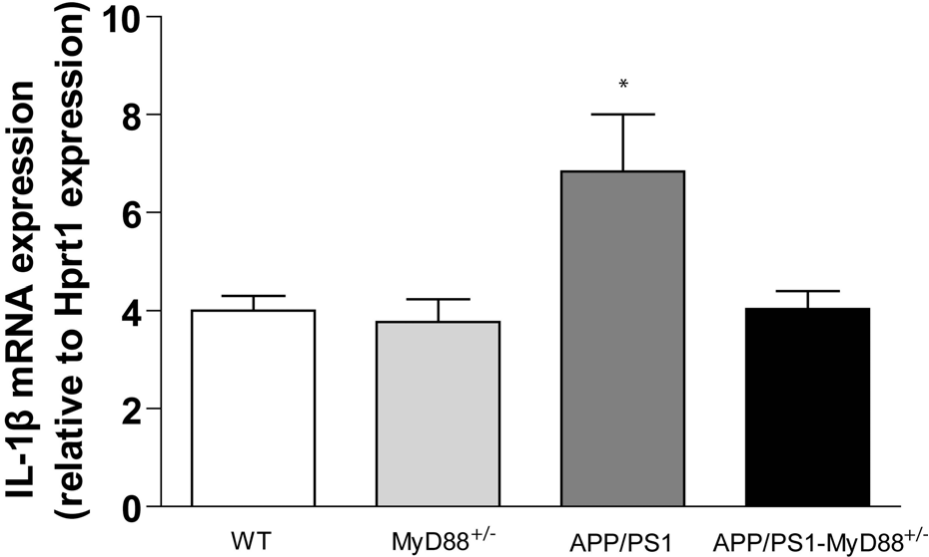
**Decreased IL-1β gene expression in the brain of controls and APP_swe_/PS1-MyD88^+/- ^mice**. Quantification of IL-1β mRNA expression by Q-PCR of 6 month-old mice revealed significant differences between the different mouse strains. Indeed, higher brain IL-1β mRNA levels were found in APP_swe_/PS1 mice compared to those measured in WT, MyD88^+/- ^and APP_swe_/PS1-MyD88^+/- ^mice. Error bars: SEM; n = 4-7; *P < 0,05 (significantly different from the other groups). (Statistical analysis was performed using one-way ANOVA followed by a Bonferroni *post-hoc *test).

We finally investigated the inflammatory status in the brains of APP_swe_/PS1-MyD88^+/- ^mice by measuring the mRNA levels of the pro-inflammatory cytokine IL-1β (Figure 5). A significant increase in IL-1β gene expression was found in the brain of APP_swe_/PS1 mice, which was essentially abolished in a context of MyD88 deficiency. IL-1β gene expression levels were significantly higher in APP_swe_/PS1 mice than all other groups of mice ((6,84 ± 1,16 vs 4,00 ± 0,30 (WT), 3,77 ± 0,47 (MyD88^+/- ^) and 4,04 ± 0,37 (APP_swe_/PS1-MyD88^+/-^)). These data suggest that MyD88 plays a very important role to regulate IL-1β gene transcriptional activation in presence of Aβ in this mouse model of AD.

## Discussion

Aβ accumulation is suggested to be a key event leading to development and progression of AD. Production of these toxic proteins is associated with neuronal apoptosis, reactive astrocytes, activated microglia and production of inflammatory molecules. Innate immune response plays a crucial role in the course of AD. However, the exact role of activated microglia is still under debate. These cells can release neurotoxic mediators or can be neuroprotective in promoting the clearance of toxic Aβ from the brain. Several lines of evidence have shown that microglia activated via TLRs can induce beneficial effects on AD progression. We have previously demonstrated that TLR2 is important to delay the cognitive decline in a mouse model of AD [21]. The role of TLR2 in microglial activation was also highlighted in the study from Jana *et al*. [20]. A small proportion of microglia in APP_swe_/PS1 mice brain expresses TLR2 mRNA, which is a reliable index of pro-inflammatory signaling in myeloid cells [20,21]. Other induced innate immune receptors in AD brains include CD14, TLR4, TLR5, TLR7 and TLR9 [9,20,25-28]. Phagocytosis of Aβ by activated microglia is significantly increased in presence of ligands that activate TLRs [11,29-31]. A functional interaction was also demonstrated between fibrillar Aβ_1-42 _peptides and the innate receptor CD14 and this interaction resulted in microglial activation [25]. Furthermore TLR2, TLR4 and CD14 are involved in fAβ phagocytosis [13]. Activation of TLR/MyD88 pathway may therefore act as a natural defense mechanism in presence of toxic Aβ and restrict disease progression.

Quantification of MyD88 mRNA revealed an upregulation of this gene in response to Aβ as depicted by a significant increase in APP_swe_/PS1 mice compared to WT. This suggests a potential function of this gene in AD. To study the role of MyD88 in AD, we used MyD88 knockout mice. Unfortunately, APP_swe_/PS1 mice deficient in MyD88 gene were not viable. This suggests that MyD88 signaling cascade acts as a natural defense mechanism to prevent Aβ toxicity in this transgenic model, even during mouse development. However it will be important in future studies to test the effects of MyD88 deficiency in other mouse models of AD, because the possibility remains that such a lethally depends on the mouse lines used in this study.

Thus, we created APP_swe_/PS1 mice heterozygous for the MyD88 gene. This model showed a significant decreased expression of the MyD88 transcript. In our previous report, we have shown that APP_swe_/PS1 mice deficient in TLR2 expression accelerated spatial and contextual memory impairments. Here, we demonstrate that a reduction in MyD88 expression also induced such behavioral impairments. Analysis of hippocampus-based spatial working learning was assessed in T-water maze. In this behavioral test, the submerged platform is the positive reinforcement. Mice were first trained to learn the position of the platform in the maze. Forty-eight hours later their reversal learning abilities were then challenged, as the submerged platform was placed at the opposite side of the maze. APP_swe_/PS1-MyD88^+/- ^mice were impaired in reversal training, as seen with a higher delay and number of errors made to reach the criterion compared to APP_swe_/PS1 mice. These data demonstrate the importance of the MyD88-dependent pathway in a context of memory impairment induced by Aβ.

Area occupied by amyloid-β plaques was calculated in the brain of APP_swe_/PS1-MyD88^+/- ^mice. We found that disease severity demonstrated by behavioral impairment in the T-water maze did not correlate well with plaque load in the brain. Indeed, APP_swe_/PS1-MyD88^+/- ^mice had fewer plaques at 6 and 9 months compared to APP_swe_/PS1 mice. Historically, accumulation of Aβ plaques was indicative of AD progression in human and mouse models. However, as mentioned above, accumulating evidence suggests that soluble and small oligomeric forms of Aβ within human brain is more closely associated with disease severity [2]. Soluble Aβ in different protein pools has deleterious effects in the brain and promotes disease progression by inducing changes in synaptic functions, behavioral deficits and promoting neuronal degeneration [32-34]. We were able to show that partial MyD88 gene deletion caused significant increases in soluble Aβ species in extracellular, intracellular and membrane-associated enriched protein pools. Nevertheless, we cannot rule out the possibility that the membrane-associated enriched proteins can also contain insoluble Aβ, because of the 3% SDS concentration used in the buffer. Although sAPPα fragment is known to be a neurotrophic factor when it is secreted in the extracellular space [35,36], Aβ has been shown to promote microtubule transport impairments in neurons and subsequently sequester sAPPα inside the cell and prevent its secretion [37]. Accordingly, sAPPα fragment levels were higher in intracellular proteins fractions of APP_swe_/PS1-MyD88^+/- ^mice while there was no significant difference in extracellular-enriched proteins level.

It is tempting to propose that senile plaques may serve as an inert reservoir of Aβ, thus protecting neurons from soluble oligomeric forms of Aβ [38]. These data regarding the plaque load are in agreement with our previous study in the APP_swe_/PS1-TLR2^-/- ^mice that had less Aβ plaques than their control APP_swe_/PS1 littermates [21]. As in APP_swe_/PS1-MyD88^+/- ^mice, memory capacities did not correlate with plaque load in the brain of APP_swe_/PS1-TLR2^-/- ^mice. TLRs and MyD88 signaling may have a more direct role on the clearance of soluble oligomeric Aβ.

Analyze of blood monocyte subsets revealed changes in APP_swe_/PS1 mice in a context of partial MyD88 deficiency. These mice exhibited significant relative decrease in Gr1^+ ^and increase in Gr1^- ^monocytes. Globally, there was a 30% reduction in the ratio of Gr1^+^/Gr1^- ^monocytes in blood of APP_swe_/PS1-MyD88^+/- ^compared to APP_swe_/PS1 mice. As explained earlier, inflammatory monocytes (CX_3_CR_1_^Lo^CCR2^+^Gr1^+^) and resident monocytes (CX_3_CR_1_^Hi^CCR2^-^Gr1^-^) seem to play opposite roles. Inflammatory monocytes are recruited quickly to inflammatory sites and differentiate into tissue specific macrophages and dendritic cells, whereas resident monocytes infiltrate tissues in an inflammation-independent fashion [24]. This process has been described in different tissues including brain in mouse models of multiple sclerosis [39] and axonal injury [40]. Moreover, inflammatory monocytes have also been shown to be the main precursors for microglial cells under inflammatory conditions of the CNS [41,42]. MCP-1 (CCL2) is a major chemiokine involved in the recruitment of monocytes in AD patients [43,44] and in mouse models of this pathology [45,46]. Since interaction between MCP-1 and its binding CCR2 receptor stimulates the mobilization of bone marrow-derived inflammatory monocytes [47], a decrease production of this chemokine may be the mechanism underlying the 30% reduction in the ratio of Gr1^+^/Gr1^- ^monocytes in blood of APP_swe_/PS1-MyD88^+/-^. In this regard, MyD88 signaling is essential for the transcriptional activation of MCP-1 in response to bacterial ligands [15,48], brain injury [40] and endogenously produced toxic proteins [14]. Moreover, MyD88-deficient mice have impaired monopoiesis during bacterial infection resulting in significant reduction in blood, splenic and bone marrow progenitors of inflammatory monocytes [49]. This suggests an important role for MyD88-mediated monocyte homeostasis under inflammatory conditions.

On the other hand, it seems that inflammatory CX_3_CR_1_^Lo^CCR2^+^Gr1^+ ^monocytes differentiate preferentially in M1-like macrophages while resident CX_3_CR_1_^Hi^CCR2^-^Gr1^- ^monocytes are likely to become M2-like macrophages [50,51]. M1 macrophages are known to have high phagocytic, proteolytic and inflammatory functions while M2 is suggested to be an anti-inflammatory subset, which is known to promote functions such as tissue remodeling, angiogenesis and matrix deposition [52]. M1-like macrophages would therefore be more efficient to clear Aβ from the brain in a context of chronic inflammation induced by accumulation of cerebral Aβ. Taking that into consideration, a decreased Gr1^+^/Gr1^- ^ratio in the pool of available monocytes of APP_swe_/PS1-MyD88^+/- ^mice may be associated with less M1-like macrophages and more M2-like cells and explain the higher levels of soluble Aβ in brain of APP_swe_/PS1-MyD88^+/- ^mice. However, such a possible mechanism has to be fully investigated.

Low IL-1β mRNA levels in the brain of APP_swe_/PS1-MyD88^+/- ^mice compared to APP_swe_/PS1 provide evidence that MyD88 is a critical molecule for the regulation of such inflammatory pathway in response to Aβ accumulation. This cytokine is crucial to orchestrate the inflammatory response by microglia [53] and a sustained production of IL-1β was reported to be beneficial by reducing Aβ pathology in a mouse model of AD [54]. We consequently believe that the innate immune response by microglia is compromised in a context of MyD88 deficiency, which may prevent their adequate activation to fight against and clear Aβ peptides. These data converge into a primary role of MyD88-signaling pathway to act as a natural protective mechanism in presence of toxic Aβ.

In summary, we show here that MyD88 plays a significant role in the evolution of AD. Partial MyD88 deficiency worsens cognitive deficit in APP_swe_/PS1 mice, as well as increases brain soluble oligomeric Aβ. Moreover, APP_swe_/PS1 mice deficient in MyD88 have reduced IL-1β gene expression and altered blood monocyte homeostasis leading to a decrease in the inflammatory population. Altogether, these results clearly demonstrate the crucial role of functional MyD88 signaling and support the importance of TLRs to prevent or delay AD pathology.

## Competing interests

The authors declare that they have no competing interests.

## Authors' contributions

JPM carried out western blots, FACS experiments and Q-PCR. KR carried out stereology analysis, behavioral tests and Q-PCR. JPM and KR performed statistical analysis and drafted the manuscript. SR conceived of the study, participated in its design and coordination and helped to draft the manuscript. All authors read and approved the final manuscript.
